# Intergenerational income mobility and psychotropic drug dispensation in a 1953 Stockholm cohort: a diagonal reference model approach

**DOI:** 10.1007/s10654-026-01425-y

**Published:** 2026-07-04

**Authors:** Klara Gurzo, Anna Oksuzyan, Bitte Modin

**Affiliations:** 1 Department of Public Health Sciences, Stockholm University, Stockholm, Sweden; 2 Center for Health Equity Studies, Stockholm, Sweden; 3 School of Public Health, Bielefeld University, Bielefeld, Germany; 4 Max Planck Institute for Demographic Research, Rostock, Germany

**Keywords:** Social mobility, Income mobility, Mental health, Psychotropic drug use, Diagonal reference model

## Abstract

The theoretical link between intergenerational social mobility and mental health has long been explored in the social sciences. Declining social mobility in high-income countries alongside rising mental health concerns have reignited interest in this research area. Sweden, while experiencing similar trends, remains a country known for its high social mobility. To investigate the relationship between social mobility and mental health, we used psychotropic drug dispensation as a proxy for mental health problems. We analyzed survey and register data of a 1953 Stockholm cohort (*n* = 11,199), followed up for psychotropic drug dispensation between ages 52–66. We measure social mobility using parental income at ages 10 and 17 and adult income between ages 37–48. Using diagonal reference models, we analyzed the association between income mobility and psychotropic drug use, stratified by gender. Adjustments were made for growing up in a single-parent household, cognitive ability, social skills, marital status, and previous hospitalization. An income gradient in psychotropic drug dispensation for the stable income group was observed, with a more pronounced pattern among men than women. Net of social origin and destination, income mobility was related to psychotropic drug dispensation among men, but not women. Downward mobility increased, while upward mobility decreased the probability of dispensing psychotropic drugs among men (0.046, *p* = 0.029 vs. − 0.047, *p* < 0.020). These results remained robust after adjustments (0.048, *p* = 0.010 vs. − 0.044, *p* < 0.017). Our findings suggest that income mobility is associated with middle-aged men’s use of psychotropic drugs. Further research is needed to confirm these results in different contexts and age groups.

**Supplementary Information:**

The online version contains supplementary material available at https://doi.org/10.1007/s10654-026-01425-y.

## Introduction

Intergenerational social mobility refers to the upward or downward movement of individuals in society’s system of social stratification relative to their parents, and has been hypothesized to impact health. An important issue to consider in this type of research is whether social mobility independently influences an individual’s health beyond the impact of childhood circumstances (social origin) and current socioeconomic position (social destination). Theoretical frameworks have suggested that any form of social mobility, whether upward or downward, may directly compromise health due to the stress (“dissociation”) associated with transitioning from familiar to unfamiliar environments [1]. An alternative theoretical proposition is that downward mobility adversely affects well-being due to feelings of failing to live up to others’ expectations (“falling from grace”) [2], whereas upward mobility may improve it via boosting a sense of self-control and exceeding expectations (“rising from rags”) [3, 4]. At the same time, the significance of social mobility in itself may pale in comparison to the influence that social origin, and especially social destination, have on health (“acculturation”) [5].

Given the hypothesis linking social mobility to health through stress processes, alongside the notable rise in the prevalence of mental health complaints in high-income countries over recent decades [6], the association between social mobility and mental health has received considerable research interest lately. Descriptive empirical findings typically suggest that the mental health of socially mobile individuals lies somewhere between the health of those they leave and those they join [7]. Earlier studies have presented descriptive patterns across different social mobility trajectories for health outcomes such as self-rated mental health [8], psychiatric disorders [9, 10], and depressive symptoms [11–13]. However, their findings are mixed. This inconsistency may, in part, arise from the limitations of conventional regression frameworks in separating the effects of social mobility from those of social origin and destination [14]. Another possible explanation may be that these studies focused on outcomes that were not as closely linked to the theorized mechanisms through which social mobility induces stress.

Recent studies have attempted to address these limitations by examining stress processes more directly and by employing more sophisticated methodologies. Various indicators have been investigated, including allostatic load [15–17], depressive symptoms [3, 18, 19] and self-rated health [20, 21], using advanced analytical techniques such as the diagonal reference model (DRM) [22, 23]. While one study found that upward mobility decreases allostatic load of younger individuals [16], contrasting findings emerged from other studies, indicating no such associations among middle-aged [17] and older individuals [15].

Studies investigating the relationship between social mobility and mental health typically assess socioeconomic position through occupational class [11, 15–18, 20] or education [3, 4]. However, income has become an increasingly preferred indicator in other areas of social stratification research [24] due to its favorable characteristics for longitudinal comparison [25]. Compared to occupational class that has traditionally dominated social mobility research, income serves as a less contextual and more objective measure [25]. Furthermore, the increasing volatility in the labor market raises questions about whether stable occupational class remains a reliable point of reference [25]. Nevertheless, studies on income mobility and health remain relatively scarce. A study conducted in Japan revealed that upward mobility negatively impacts self-rated health among men [26]. Conversely, a Canadian study found that upward income mobility correlates positively with men’s self-rated health, while downward mobility is associated with poorer self-rated health in both men and women [21]. Yet another Canadian study failed to identify any significant association between income mobility and symptoms of psychological distress [19].

The interplay of social mobility, health, and gender is somewhat ambiguous as well. Notably, the health gradient by socioeconomic position tends to be steeper for men compared to women, particularly concerning income [27]. This suggests that social mobility may operate through distinct mechanisms, yielding varying health effects for men and women. Although gender-specific analyses have only recently become common practice, many studies do not find any significant association between social mobility and mental health for neither men nor women [7, 15–17, 19, 20]. In instances where social mobility effects are detected, they often prove significant for men only [3, 9, 10, 13, 21] or there are no systematic differences between men and women [12, 21].

Psychotropic drug use, reflects a wide range of mental ill health symptoms since these drugs are prescribed from mild to severe mental health problems [28]. They are prescribed for multiple mental health disorders, and their prevalence has been on the rise in recent decades [29]. Furthermore, while studies of mental health and well-being often rely on self-reported information, which tend to suffer from underreporting [30], register information on psychotropic drug dispensation covers the entire population. Despite these favorable characteristics, we do not know of any study that has analyzed it in relation to social mobility.

Recent social mobility research shows a decline in upward mobility and a rise in downward mobility, with Nordic countries losing their status as exceptionally fluid societies [31]. In particular, there has been a decline in income mobility in Sweden [32]. These shifts in mobility, coupled with the rise in psychotropic drug prescriptions over recent decades [29], make the relationship between income mobility and psychotropic drug intake worth examining. This study therefore explores intergenerational income mobility and psychotropic drug dispensation within a Swedish cohort born in 1953 through DRM, controlling for relevant confounders.

Utilizing a unique Swedish data set combining linked register and survey data, we can consider several confounding factors. Previous analyses have shown a more pronounced mortality gradient by income among men than women in this cohort [33]. Therefore, we investigate the association between income mobility and mental health separately for men and women. Moreover, given that individual health outcomes often exhibit gendered patterns influenced by spousal socioeconomic position [27] we control for marital status in our analysis. Growing up in a single parent household has also been identified as a factor linked to both social mobility [34] and adult health [35]. Therefore, we include this variable in our examination. Finally, we account for the bidirectional relationship between income and health in two ways. Firstly, personal characteristics are hypothesized to shape both social mobility and health prospects. Additionally, an individual’s health status may either support or hinder their income mobility and vice versa [36]. We address these factors by adjusting our analysis for childhood cognitive ability and social skills as well as psychiatric and somatic hospitalization occurring during a three-year period before we begin observing adult income.

## Data and methods

### Study population

**Fig. 1:**
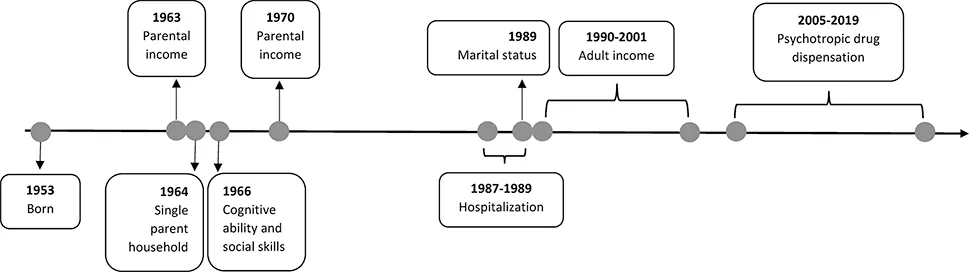
Structure of the data set

The data for this study came from the Stockholm Birth Cohort Multigenerational Study (SBC Multigen). This prospective cohort study linked survey data of children born in 1953 and residing in the Stockholm area in 1963 (*N* = 15,117) with register data for the cohort members and their parents [37]. The study population comprised SBC Multigen cohort members residing in Sweden at the beginning of the follow-up on 1 July 2005 (*n* = 13,409). The final analytical sample consisted of 11,199 individuals who had complete data on the variables used in the analyses. Figure 1 described the structure of our data set.

### Variables

**Independent variable**: Mobility is measured through the change between parental and own adult income. Parental income information was obtained from the Register of Population and Income in 1963 and 1970 (when the cohort members were 10 and 17 years old). It was measured through the combined average pretax labor income of both parents in both years. Information on adult income was collected from LISA (Longitudinal Integrated Database for Health Insurance and Labor Market Studies) and defined as the average pretax labor income all years between 1990 and 2001 (between the ages of 37 and 48). Income measures were inflation-adjusted in 2001 SEK. The income distribution was calculated separately for women and men. Mobility was categorized into three groups, indicating whether individuals moved up, moved down, or remained in the same income tertile as their parents.

**Outcome variable**: Information on psychotropic medication dispensation was derived from the Prescribed Drug Register, which includes all medications prescribed by physicians and dispensed at pharmacies. We use the term psychotropic drug ‘dispensation’ instead of ‘intake’ since we only know that the drug was collected from the pharmacy with a prescription, but not whether it was taken. The outcome measure was dichotomized as having any psychotropic drug dispensation based on the first prescription between July 2005 and December 2019 (between the ages of 52 and 66). The following categories from the Anatomical Therapeutic Chemical (ATC) classification system were included: neuroleptics (N05A), anxiolytics (N05B), hypnotics and sedatives (N05C), and antidepressants (N06A).

**Control variables**: Single-parent household information was obtained from the Register of Population and Income in 1964 (age 11 or 12). Cognitive ability was measured in 1966 through a cognitive test including a verbal, spatial, and numerical part (age 13 or 14). A sociometric assessment also took place in the same year, where students were asked to nominate three of their classmates as best friends. The number of nominations that the cohort member received was used as a proxy for social skills. Marital status was a categorical variable retrieved from the 1989 Total Population Register (age 36). Information on psychiatric and somatic hospitalization was retrieved from the Swedish National Patient Register. Since full coverage of this register was not attained until 1987, we consider hospitalization between 1987 and 1989, the years before we start observing adult income (ages of 34–36). Hospitalization was a binary variable indicating whether or not the cohort member had been hospitalized, excluding diagnoses related to pregnancy or childbirth (ICD-9 630–679) and factors influencing health status and contact with health services (V000-V69).^1^

### Statistical analysis

Traditional regression models used in the context of social mobility face a challenge due to linear dependency between positions of origin and destination. Researchers often seek to measure the influence of socioeconomic position at various life stages by simultaneously controlling for these factors. However, the close relationship between these variables leads to collinearity problems when included in the same model. Additionally, the derived social mobility variable, whether as an interaction term (Square Additive Model [38]) or as a categorical variable (Difference Method) blends effects from both mobile and stable groups when estimating ‘mobility effects’ [39].

DRM seeks to address these issues by distinctly separating the mobile and stable groups [22, 23]. Therefore, we applied it to estimate the association of income mobility with psychotropic drug dispensation. Conceptually, DRM considers the mobility matrix, where the rows refer to position of origin and the columns to destination. The model calculates the averages of the stable groups in the diagonal cells, with a weight parameter expressing the relative salience of origin and destination in determining the outcome. Then the weighted means are taken in the off-diagonal cells and are compared to the diagonal cells corresponding to the specific origin and destination. This allows for the avoidance of linearly confusing effects of origin and destination from mobility.

Formally, the DRM can be applied to our case in the following way.

$$\:{Y}_{ijk}=\:w*{\mu\:}_{ii}+\left(1-w\right){\mu\:}_{jj}+\:{\beta\:M}_{ij}+\:\sum\:{\gamma\:x}_{ijk}+{e}_{ijk}$$

Here, $\:{Y}_{ijk}$ is psychotropic drug dispensation for individual *k* in social origin (parental income) * i* and destination (adult income) *j*. Parameters $\:{\mu\:}_{ii}$ and $\:{\mu\:}_{jj}$ refer to the estimated mean of *Y* among the stable groups in the diagonal cells. Although not shown explicitly in the equation, the model includes a single constant representing the overall mean level of psychotropic drug dispensation; the diagonal group parameters are estimated as deviations from this constant. The diagonal group means define the reference structure of the model, while the weight parameter *w* captures the relative contribution of origin versus destination among mobile individuals. *W* constrained between 0 and 1 corresponds to the position of origin, while 1-*w* refers to the position of destination. A *w*-value higher than 0.5 implies that, among mobile individuals, outcomes more closely resemble those of the stable origin group, whereas a *w*-value less than 0.5 implies closer resemblance to the stable destination group.

Importantly, the diagonal reference model does not impose a linear association between income mobility and psychotropic drug dispensation. Rather than treating mobility as a continuous exposure, expected outcomes for mobile individuals are defined by their origin and destination income positions. Any additional effect of mobility is assessed as a deviation from this origin–destination–based expectation. We therefore included a categorical mobility variable *M* capturing upward mobility, downward mobility, and non-mobility to assess whether mobile individuals deviated systematically from the outcomes expected given their origin and destination income positions. Finally, we further extend this model by controlling for a vector of variables $\:{\gamma\:x}_{ijk}$, which includes factors such as living in a single-parent household, cognitive ability and social skills during childhood, as well as marital status and hospitalization history (age 34–37 years). To account for gender differences in psychotropic drug use and the impact of income position, the analysis was conducted separately for men and women. We used DRMs with a linear link function (linear probability model), and compared the fit of the adjusted models with the base model using the Akaike Information Criteria (AIC) and Bayesian Information Criteria (BIC). The analysis was performed using Stata 18 and DRM estimates were derived using the “drm” module [40].

## Results

The study population consisted of 11,199 individuals, with 53% being women (see Table 1 for descriptive statistics). Men had, on average, higher income and marginally higher scores on cognitive ability and social skills than women. Conversely, women were more likely to have lived in single-parent households during childhood, been married or divorced, experienced hospitalization between ages 34–36, and of having utilized psychotropic drugs than men. The proportion of individuals using psychotropic drugs in our sample is higher than the official yearly national average, partly because we captured any dispensation over a 14-year period, and partly because dispensation rates are, on average, higher in Stockholm compared to the rest of the country [41].

**Table 1:** Descriptive statistics (*n* = 11,199)

	Women (n = 5,903)	Men (n = 5,296)
Independent variables	Mean (SD)	Mean (SD)
Parental income^a, b, c^ (ages 10, 17)		
First tertile	94,399 (29,274.2)	97,648 (28,759.3)
Second tertile	152,073 (14,282.2)	152,890 (13,563.1)
Third tertile	263,541 (117,382.6)	262,721.8 (117,482.4)
Adult income^a, c^ (ages 37–48)		
First tertile	90,089 (45,262.3)	120,758 (64,352.9)
Second tertile	180,560 (16,287.1)	253,090 (27,261.2)
Third tertile	276,404 (84,806.8)	451,673 (209,191.1)
*Control variables*		
Cognitive ability (age 13)	67.4 (17.7)	71.5 (16.7)
Social skills^e^ (age 13)	2.5 (1.7)	2.8 (1.8)
	Freq (%)	Freq (%)
Living in single parent household (age 11)	546 (9.3)	441 (8.3)
Marital status (age 36)		
Married	3582 (60.7)	2867 (54.14)
Not married	1677 (28.4)	2057 (38.8)
Divorced	622 (10.5)	369 (7.0)
Widowed	22 (0.4)	3 (0.1)
Any hospitalization (ages 34–36)	821 (13.9)	535 (10.1)
Any hospitalization (ages 19–36)^f^	3035 (51.4)	2199 (41.5)
Any hospitalization (ages 16–36)^f^	3175 (53.8)	2329 (44.00)
*Outcome variable*		
Any psychotropic drug dispensation (ages 52–66)	3223 (60.9)	2065 (39.0)
N05A neuroleptics	246 (4.7)	328 (5.6)
N05B anxiolytics	1191 (20.2)	653 (12.3)
N05C sedatives	2379 (40.3)	1514 (28.6)
N06A antidepressants	1953 (33.1)	1159 (21.9)

^a^ Income measures expressed in 2001 SEK; ^b^ Both mother’s and father’s income; ^c^ Mean income within tertile ^d^ Ages when parental income of cohort member is measured ^e^ Measured by sociometric popularity (total received friendship nominations) ^f^ Used only in the sensitivity analysis

Approximately 40% of both women and men fell into the stable mobility category by remaining in the same income tertile as their parents (Table 2). Psychotropic drug dispensation was most prevalent in the stable low-income category for both women (62%) and men (49%) (Table 3). Conversely, the lowest prevalence rates were observed in the stable high-income category for women (49%) and the upwardly mobile groups for men, transitioning from the middle-income to the high-income tertile (31%).

**Table 2:** Transition matrices by gender (n=11,199)

	Women (*n* = 5,903) Frequency %			Men (*n* = 5,296) Frequency %		
Parental income	Adult income					
	Low	Middle	High	Low	Middle	High
Low	768	683	517	694	629	443
	(13.0)	(11.6)	(8.8)	(13.1)	(11.9)	(8.4)
Middle	645	721	602	592	644	529
	(10.9)	(12.2)	(10.2)	(11.9)	(12.1)	(9.9)
High	555	564	848	480	492	793
	(9.4)	(9.6)	(14.4)	(9.0)	(9.3)	(14.9)

**Table 3:** Frequency of psychotropic drug dispensation by social mobility trajectories (n=11,199)

	Women (*n* = 5,903) %			Men (*n* = 5,296) %		
Parental income	Adult income					
	Low	Middle	High	Low	Middle	High
Low	62.2	53.4	54.0	49.0	37.7	33.0
Middle	60.0	52.1	50.7	44.1	33.9	30.8
High	54.8	55.0	49.4	47.9	40.2	34.3

Table 4 presents the results of the DRMs, where estimates for the stable income groups indicate higher or lower levels of psychotropic drug dispensation relative to the model’s reference level (the constant). For women, a stable low income was associated with higher-than-average likelihood of psychotropic drug dispensation, whereas stable high income was linked to lower-than-average likelihood across all models. Income weights in the unadjusted models indicated that parental income was less influential than adult income in determining psychotropic drug dispensation among mobile women (0.300, *p* < 0.005 vs. 0.700, *p* < 0.001). However, after accounting for income mobility, the estimated weights among women were associated with large uncertainty and could not be interpreted as clearly favoring either origin or destination relative to equal salience (0.5). Furthermore, the mobility estimates were not significantly associated with psychotropic drug dispensation in any of the models. Accordingly, model fit statistics did not improve after adjusting for income mobility.

**Table 4:** Psychotropic drug dispensation (2005-2019, ages 52–66) by social mobility (n=11,199) – Diagonal reference model with linear link

	Women (*N* = 5,903)			Men (*N* = 5,296)		
	Base model	Mobility adjusted	Fully adjusted	Base model	Mobility adjusted	Fully adjusted
Stable low income	0.066***	0.063***	0.045**	0.083***	0.091***	0.069***
	(0.012)	(0.013)	(0.014)	(0.014)	(0.013)	(0.013)
Stable middle income	− 0.017	− 0.010	− 0.007	− 0.022	− 0.046***	− 0.046***
	(0.012)	(0.015)	(0.016)	(0.012)	(0.014)	(0.013)
Stable high income	− 0.048***	− 0.053***	− 0.038**	− 0.062***	− 0.046***	− 0.023
	(0.012)	(0.012)	(0.013)	(0.01)	(0.013)	(0.014)
Weights^a^						
Parental income (ages 10, 17)	0.300**	0.764	0.831	0.034	0.474***	0.538***
	(0.107)	(0.390)	(0.598)	(0.117)	(0.142)	(0.145)
Adult income (ages 37–48)	0.700***	0.236	0.169	0.966***	0.526***	0.462**
	(0.107)	(0.390)	(0.598)	(0.117)	(0.142)	(0.145)
Social mobility (ref = stable)						
Downward		0.040	0.036		0.046*	0.048*
		(0.033)	(0.035)		(0.021)	(0.019)
Upward		− 0.040	− 0.033		− 0.047*	− 0.044*
		(0.033)	(0.036)		(0.020)	(0.018)
Single parent household (age 11)			0.008			0.016
			(0.022)			(0.024)
Cognitive ability (age 13)			− 0.001*			− 0.001*
			(0.000)			(0.0004)
Social skills (age 13)			− 0.002			− 0.007
			(0.004)			(0.004)
Marital status (age 36) (ref = married)						
Not married			0.005			0.007
			(0.015)			(0.014)
Divorced			0.063**			0.065*
			(0.021)			(0.027)
Widowed			0.151			− 0.071
			(0.105)			(0.278)
Any hospitalization (ages 34–36)			0.177***			0.146***
			(0.019)			(0.022)
Constant	0.556***	0.555***	0.567***	0.39***	0.391***	0.452***
	(0.007)	(0.010)	(0.029)	(0.007)	(0.011)	(0.034)
AIC	8493.753	8495.159	8400.269	7355.171	7354.271	7307.825
BIC	8527.169	8541.942	8493.834	7388.044	7400.294	7399.871

^*a*^An estimate for parental income weight larger than 0.5 indicates origin is more salient than destination among mobile individuals. *Standard errors are in parentheses*, *** p<0.001, ** p<0.010, * p<0.050

For men, stable low income was associated with an increased likelihood of psychotropic drug dispensation, whereas stable middle and stable high income were linked to lower-than-average dispensation across all models. The weight parameters in the unadjusted model showed that parental income had little influence on psychotropic drug dispensation among mobile men (0.034, *p* < 0.770), whereas adult income was highly influential and statistically significant. (0.966, *p* < 0.001). After adjusting for income mobility, the estimated weight for parental income increased substantially and approached that of adult income (0.474, *p* < 0.001 vs. 0.526, *p* < 0.001). In the fully adjusted model, the estimated weight placed slightly more emphasis on parental income than on adult income (0.538 vs. 0.462) among mobile men.

Income mobility showed a significant association with psychotropic drug dispensation among men: downward mobility increased dispensation by 4.6% (*p* = 0.029), while upward mobility decreased dispensation by 4.7% (*p* < 0.020), compared to those whose income remained stable. These results remained robust even after full adjustment (downward: 4.8%, *p* = 0.010; upward: 4.4%, *p* < 0.017). Controlling for income mobility improved AIC slightly, while BIC did not improve.

A total of 820 cohort members died during the follow-up period, which may have influenced our results. It is likely that many of these individuals experienced compromised health prior to death, which could have affected their ability to maintain or improve their income. In such cases, downward mobility may reflect the consequences of deteriorating health rather than a factor contributing to it. Furthermore, if these individuals also received psychotropic medications before death, their drug use might be wrongly interpreted as a result of downward mobility, when in fact, both outcomes may stem from underlying health problems. To address this potential source of bias, we excluded deceased individuals and reran the analysis. The results were very similar to those obtained from the full sample (Supplementary Table S1).

For some cohort members, the registered labor income was zero either in adulthood or childhood. Having no registered labor income might be due to various reasons (nontaxable incomes, tax evasion, household work, inactivity in the labor market due to health problems or functional disabilities, etc.) and such individuals are likely qualitatively different from other low-income earners. We therefore excluded this group from our sample and re-estimated the models. Once again, the results were very similar to our main findings (Supplementary Table S2).

In addition to adjusting our analysis for hospitalization between 1987 and 1989, we tested the robustness of our results by including controls for hospitalization over longer periods. To account for health selection in early adulthood, we utilized the earliest available data. Hospitalization records, covering Stockholm but not all Swedish regions, are available from 1969. However, most regions reported hospitalizations from somatic (but not mental) diagnoses from 1972. We created two binary variables with observation windows starting in 1969 or 1972, respectively, and ending in 1989. Our results remained significant even when these long periods were controlled for hospitalization (Supplementary Table S3). For men, social mobility estimates were only slightly attenuated.

We applied linear regression within our DRMs to facilitate easier interpretation. However, since logistic regression is a more commonly used method for modeling binary outcomes, we reran the main analysis accordingly. The results from the logistic and linear models were highly similar (Supplementary Table S4).

Finally, we extended the analysis to the full 1953 Swedish birth cohort to enhance the generalizability of our findings. For parental income, we used the average of pretax labor income from 1970 to 1975, which were the earliest years available in the registers. For adult income, the same variables as in the main analysis were used. Among the control variables, only hospitalization and marital status were available in the registers. When rerunning the analysis with these variables, the social mobility estimates remained statistically significant and pointed in the same directions as in the main results. However, the magnitude of these estimates was notably smaller, and the estimated weight parameters fell outside expected bounds, with adult income weights exceeding a value of one (Supplementary Table S5).

## Discussion

This study explored the relationship between intergenerational income mobility and psychotropic drug dispensation between ages 52–66 in a Swedish cohort through DRM. We observed a clear income gradient in psychotropic drug dispensation in the overall sample, which was also evident among individuals who remained in the same income tertile as their parents. Social mobility was associated with our indicator of mental health problems only for men: downward mobility increased, while upward mobility decreased, the likelihood of psychotropic drug dispensation. These results remained robust when adjusting for several confounders measured at relevant stages of the life course.

Previous research on intergenerational social mobility and mental health has produced mixed findings, with many studies reporting weak or inconsistent associations once social origin and destination are taken into account [9, 13, 15, 17, 18, 20]. Much of this literature has focused on educational or occupational mobility and relied on self-reported mental health outcomes, often measured earlier in adulthood [3, 9, 13, 20]. Fewer studies have examined income mobility [19, 21], and, to our knowledge, none have considered later-life mental health using register-based indicators of treated illness. By analyzing intergenerational income mobility in relation to psychotropic drug dispensation between ages 52 and 66, our findings extend existing work in several ways. They capture clinically recognized mental health problems over a long follow-up period, examine mobility effects at a later life stage, and highlight marked gender differences in how income mobility relates to mental health. The following paragraphs situate these findings in relation to proposed psychosocial mechanisms and prior empirical evidence.

Our main results provide support for the notion that social mobility, beyond social origin and destination, have an impact on men’s mental health in our cohort. Men who experienced downward intergenerational income mobility had a significantly higher likelihood of psychotropic drug dispensation compared to the stable income group. This aligns with the ‘falling from grace’ hypothesis, suggesting that descent in the social hierarchy vis-à-vis the parental generation triggers feelings of loss, failure and unmet expectations, potentially resulting in mental health problems [18]. Such distress, particularly prevalent in eras marked by high societal expectations for success [2], appears to have been present in the male half of this 1953 Swedish cohort.

The ethos of the times during which the 1953 cohort entered adulthood was marked by a widespread conscientiousness, characterized by the belief that knowledge, education, and hard work could improve one’s life chances [42]. Given the expansion of welfare services and the availability of housing and educational opportunities at the time [43], there may have been a stronger emphasis on individual effort rather than environmental factors. In such a context, socioeconomic failure was likely to be perceived as a personal shortcoming rather than the result of structural conditions.

Men who experienced upward intergenerational income mobility, on the other hand, displayed a significantly lower likelihood of psychotropic drug use compared to the stable income group, according to our main results. This aligns with the ‘rising from rags’ hypothesis, bolstering self-confidence, a sense of control, and gratitude [3]. Previous research has also shown that upwardly mobile individuals tend to adopt healthier behaviors associated with their new social position [44].

A previous study based on the 1953 Stockholm Birth Cohort has shown that downwardly mobile men are more prone to psychiatric hospitalization at age 30, particularly as a result of substance misuse, compared to their stable or upwardly mobile counterparts [9]. Together with our findings, these results imply that the stress caused by downward mobility might prevail at least up until middle age among men. Although the earlier study did not find any associations with upward mobility, our research indicates a positive relationship with mental health. This suggests that while upward mobility may introduce stress earlier in life, its beneficial effects on mental health likely outweigh any initial challenges. Subsequently, as individuals progress through life, these positive effects may become more pronounced.

This interpretation aligns with a German study on educational mobility, which noted no immediate impact of upward mobility on life satisfaction in early adulthood (ages 30–39). However, the study observed positive effects beginning at age forty and continuing into individuals’ sixties, as demonstrated using the DRM [45]. Another study, based on the total Swedish population, discovered that upward mobility decreased, and downward mobility increased, the likelihood of psychiatric hospitalization among 21–56-year old men, thus supporting the findings of our study [10]. However, that study did not provide age-specific mobility trajectory results, making it difficult to draw more specific conclusions about their similarity to ours. Another study, using data from ten European countries, found a relationship between downward mobility and upper aerodigestive tract cancer for men [46]. The authors argued that the remaining association after adjustments for behavioral risk factors are likely due to stress caused by psychosocial mechanisms. Finally, the only study known to us that directly examined the relationship between income mobility and self-reported psychological distress, was conducted in Canada among individuals aged 25 to 53 [19]. Utilizing DRM, this study found no intergenerational mobility effects.

Consistent with prior research, we found no discernible relationship between intergenerational income mobility and mental health problems among women [3, 9, 10, 13, 21]. Although an income gradient was observed within the non-mobile group, the relative importance of parental and adult income did not differ in relation to psychotropic drug dispensation of socially mobile women.

In general, unlike men, women’s health tend to exhibit less reliance on their income [27], a trend previously noted within this cohort as well [33]. Therefore, social mobility might not be as important for women’s health as other important aspects of life. Supporting this proposition, we found that while divorce and prior hospitalization were significant predictors of women’s psychotropic drug use, social mobility was not. However, income mobility has started to decline in recent decades, especially among women, likely as a consequence of their income more accurately reflecting their human capital [32]. In other words, women have begun to earn wages that better match their skills and abilities than they used to. This shift might also impact how social mobility influences the mental health of women. Therefore, focusing on gender differences in younger cohorts remains important.

While psychotropic medication dispensing is a well-established indicator of clinically recognized and treated mental ill-health, it does not capture the full spectrum of psychological distress in the population. Many individuals with substantial symptoms do not seek care or receive a psychiatric diagnosis, meaning that dispensing data reflect only the treated portion of overall need [47]. In addition, some psychotropic medications are prescribed for non-psychiatric indications, such as hypnotics and sedatives for sleep disturbances and certain antidepressants for chronic pain or headache [48, 49]. Because off-label prescribing practices differ across psychotropic drug classes, the presence of similar mobility-related patterns across anxiolytics, hypnotics and sedatives, and antidepressants (Supplementary Table S6) makes it unlikely that the observed associations are driven by off-label use. Moreover, anxiolytics are subject to relatively restrictive prescribing recommendations in Sweden, further reducing the likelihood that these findings are explained by non-psychiatric prescribing [50, 51].

As a final sensitivity analysis, we conducted stratified linear analyses by parental and adult income to relax the structural assumptions of the diagonal reference models, particularly the absence of an assumed linear association between mobility and the outcome. These analyses showed that adult income largely accounted for the overall gradient in psychotropic drug use, whereas parental income contributed little once adult income was fixed (Supplementary Table S7). The stratified linear analyses capture outcome differences across income strata and therefore primarily reflect income-related gradients that combine mobile and non-mobile individuals within each stratum. The diagonal reference models, by contrast, explicitly contrast mobile individuals with non-mobile reference groups defined by origin and destination income positions, assessing whether mobile individuals deviate from what would be expected given these gradients. In summary, this sensitivity analysis helps separate the strong destination-related gradient in psychotropic drug use from mobility-related deviations assessed in the DRM, thereby providing context for why mobility effects may be present even when outcome levels are dominated by adult income.

### Strengths and limitations

This study utilized high-quality register data combined with unique survey information from childhood of a Stockholm cohort born in 1953. We used psychotropic drug dispensation as an indicator of mental health problems, which we believe is a reliable proxy as they are commonly prescribed to treat a broad spectrum of mental health conditions, and less sensitive to ‘underreporting’ [30].

To mitigate attenuation bias resulting from income measurement error, we used parental and adult income measures averaged across multiple years. To reduce life cycle bias, stemming from measuring income at an age not representative of life-time income, adult income was measured around middle age [52]. By using the income of both parents and combining them before relativizing them, we attempted to measure material resources that were available to the cohort members during upbringing. For adult income, we focused solely on the individual’s own earnings rather than household income in order to capture the cohort members’ personal income generating capacity.

Furthermore, we were able to control for a wide range of important confounders at relevant stages of the life course in our analyses. Most of the control variables were significantly associated with psychotropic drug dispensation in the expected direction. Moreover, our results proved to be robust against excluding zero income observations. In order to address the likely path from health to social mobility [36], we not only reran the analysis on those who survived the follow-up period, but also controlled for prior hospitalizations across different time periods. While all forms of previous hospitalization were significantly and strongly related to psychotropic drug use, the association between income mobility and mental health problems remained statistically significant after adjusting for all forms of previous hospitalization.

Finally, we adjusted our analysis for marital status, as it may influence access to resources – either enhancing or limiting them – which can, in turn, affect both social mobility and mental health. At the same time, marital status may also act as a mediator if, for example, changes in social mobility lead to changes in marital status, which then influence mental health. To address its potential confounding role, we measured marital status prior to the period in which income mobility was assessed. Additionally, we reran the analysis without adjusting for marital status to explore its possible mediating role. This had little effect on our estimates (data not shown).

Using the SBC Multigen data allowed us to control for important, yet rarely available, confounders such as cognitive ability and social skills. However, since this sample includes only individuals born in Stockholm, the findings may be less generalizable to the broader Swedish population. To address this, we replicated our analysis using the complete 1953 Swedish cohort. In the results based on this full sample, the weight parameters were outside the [0,1] boundaries, with adult income weight exceeding one. This may reflect the fact that parental income was measured at ages less optimal for capturing the resources available during childhood. Similarly, the national estimates were considerably smaller in magnitude compared to those from Stockholm. This difference may stem from the lower quality of the parental income measure, as well as from the higher prevalence of psychotropic drug use in Stockholm compared to the rest of the country [41]. Nevertheless, the national estimates were statistically significant and pointed in the same direction as our main findings, suggesting that our results are likely generalizable beyond Stockholm.

We applied Diagonal Reference Models to estimate the effects of social mobility while accounting for social origin and destination, which is an approach that is widely used in mobility research. Diagonal reference models have been criticized for potentially yielding null mobility effects when mobility operates primarily through linear gradients in destination status rather than through deviations from origin–destination reference positions [53]. While this focus on stable origin–destination reference groups can be seen as a strength, by restricting comparisons to individuals who remain in the same income group, it may not hold in highly mobile societies, where norms and values of stable groups can be influenced by the mobile population [54]. DRM also has limitations in causal inference, as it does not fully account for non-random selection into mobility. Nevertheless, the lack of a clear counterfactual for mobility applies to most micro-level studies. While our findings are descriptive rather than causal, DRM enables us to contribute to the ongoing research on social mobility by providing meaningful empirical patterns.

### Conclusions

Based on a Stockholm cohort born in 1953, this study offers cautious evidence that income mobility, independent of social origin and destination, is related to psychotropic drug use among men. While downward mobility increased this likelihood, upward mobility decreased it. Interestingly, while women exhibited a higher likelihood of psychotropic drug dispensation than men, their usage remained unaffected by income mobility. Further investigation is warranted to understand the reasons behind this contrast. The robustness of our results was confirmed after adjusting for various confounding factors, with consistent patterns observed in the full Swedish 1953 birth cohort. Future research could explore the relationship between social mobility and psychotropic drug use across various contexts and applying diverse socioeconomic measures.

## Supplementary Information

Below is the link to the electronic supplementary material.

Supplementary Material 1

## Data Availability

The authors do not have permission to share data.

## Footnotes

1 Other codes excluded due to being similar diagnoses: 3009, 3748, 4788, 5188, 5308, 5642, 5694, 5698, 5758, 5778, 5938, 5968, 6028, 6078, 6088, 6118, 6208, 6218, 6228, 6594, 6595, 6598, 7360, 7362, 7367, 7368, 7369, 7380, 7383, 7608, 7958, V51.

## Declarations

### Generative AI and AI-assisted technologies in the writing process

During the preparation of this work the authors used ChatGPT in order to improve the language and readability of the text. After using this tool/service, the authors reviewed and edited the content as needed and take full responsibility for the content of the publication.
