# Supplementary material for: Intergenerational income mobility and psychotropic drug dispensation in a 1953 Stockholm cohort: a diagonal reference model approach
Source: Eur J Epidemiol. 2026 Jul 4;41(8):993–1003. doi: 10.1007/s10654-026-01425-y (PMC13612693; doi:10.1007/s10654-026-01425-y)
Supplement: Supplementary file 1 [file 10654_2026_1425_MOESM1_ESM.docx]

Supplementary Table S1 Psychotropic drug dispensation (2005-2019, ages 52-66) by social mobility (n=10,379) excluding cohort members who died during the follow-up - Diagonal reference model with linear link

|  | Women (N=5,553) | | | Men (N=4,826) | | |
| --- | --- | --- | --- | --- | --- | --- |
|  | Base model | Mobility adjusted | Fully adjusted | Base model | Mobility adjusted | Fully adjusted |
| Stable low income | .063*** | .062*** | .046*** | .061*** | .072*** | .057*** |
|  | (.013) | (.013) | (.013) | (.015) | (.013) | (.014) |
| Stable middle income | -.015 | -.012 | -.009 | -.009 | -.036** | -.037** |
|  | (.012) | (.014) | (.014) | (.011) | (.014) | (.013) |
| Stable high income | -.048*** | -.050*** | -.037** | -.052*** | -.036** | -.019 |
|  | (.012) | (.013) | (.013) | (.011) | (.013) | (.014) |
| Weights^a^ |  |  |  |  |  |  |
| Parental income (ages 10, 17) | .316** | .691* | .695 | -.053 | .558** | .626*** |
|  | (.114) | (.345) | (.450) | (.18) | (.183) | (.186) |
| Adult income (ages 37-48) | .684*** | .309 | .305 | 1.053*** | .442* | .374* |
|  | (.114) | (.345) | (.450) | (.18) | (.183) | (.186) |
| Social mobility (ref = stable) | | |  |  |  |  |
| Downward |  | .033 | .028 |  | .047* | .049* |
|  |  | (.030) | (.029) |  | (.022) | (.019) |
| Upward |  | -.031 | -.023 |  | -.045* | -.043* |
|  |  | (.030) | (.030) |  | (.021) | (.019) |
| Single parent household (age 11) | |  | .008 |  |  | .013 |
|  |  |  | (.023) |  |  | (.025) |
| Cognitive ability (age 13) |  |  | -.001 |  |  | -.001 |
|  |  |  | (.000) |  |  | (.000) |
| Social skills (age 13) |  |  | -.002 |  |  | -.007 |
|  |  |  | (.004) |  |  | (.004) |
| Marital status (age 36) (ref = married) | | |  |  |  |  |
| Not married |  |  | -.002 |  |  | -.008 |
|  |  |  | (.015) |  |  | (.015) |
| Divorced |  |  | .063** |  |  | .050 |
|  |  |  | (.022) |  |  | (.029) |
| Widowed |  |  | .189 |  |  | -.058 |
|  |  |  | (.108) |  |  | (.276) |
| Any hospitalization (ages 34-36) | |  | .177*** |  |  | .124*** |
|  |  |  | (.020) |  |  | (.024) |
| Constant | .535*** | .535*** | .548*** | .368*** | .368*** | .430*** |
|  | (.007) | (.011) | (.030) | (.007) | (.011) | (.035) |
| AIC | 8013.538 | 8015.722 | 7933.152 | 6607.163 | 6608.021 | 6583.533 |
| BIC | 8046.649 | 8062.076 | 8025.861 | 6639.572 | 6653.394 | 6674.278 |
| *^a^*An estimate for parental income weight larger than .5 indicates origin is more salient than destination among mobile individuals. *Standard errors are in parentheses, *** p<.001, ** p<.010, * p<.050* | | | | | | |

Supplementary Table S2 Psychotropic drug dispensation (2005-2019) by social mobility after excluding zero income groups (n=10,974) – Diagonal reference model with linear link

|  | Women (N=5,786) | | | Men (N=5,188) | | |
| --- | --- | --- | --- | --- | --- | --- |
|  | Base model | Mobility adjusted | Fully adjusted | Base model | Mobility adjusted | Fully adjusted |
| Stable low income | .064*** | .059*** | .044** | .075*** | .085*** | .065*** |
|  | (.013) | (.014) | (.014) | (.014) | (.013) | (.014) |
| Stable middle income | -.017 | -.007 | -.004 | -.015 | -.042** | -.043*** |
|  | (.012) | (.013) | (.012) | (.011) | (.014) | (.013) |
| Stable high income | -.046*** | -.052*** | -.039*** | -.059*** | -.043** | -.022 |
|  | (.012) | (.012) | (.012) | (.01) | (.013) | (.014) |
| Weights^a^ |  |  |  |  |  |  |
| Parental income (ages 10, 17) | .326** | .903* | .990 | -.015 | .487** | .560*** |
|  | (.111) | (.426) | (.583) | (.138) | (.158) | (.155) |
| Adult income (ages 37-48) | .674*** | .097 | .010 | 1.015*** | .513** | .440** |
|  | (.111) | (.426) | (.583) | (.138) | (.158) | (.155) |
| Social mobility (ref = stable) | | |  |  |  |  |
| Downward |  | .046 | .042 |  | .046* | .048* |
|  |  | (.034) | (.034) |  | (.022) | (.019) |
| Upward |  | -.049 | -.042 |  | -.047* | -.044* |
|  |  | (.034) | (.034) |  | (.021) | (.018) |
| Single parent household (age 11) | |  | .008 |  |  | .017 |
|  |  |  | (.023) |  |  | (.024) |
| Cognitive ability (age 13) |  |  | -.001 |  |  | -.001* |
|  |  |  | (.000) |  |  | (.000) |
| Social skills (age 13) |  |  | -.001 |  |  | -.007 |
|  |  |  | (.004) |  |  | (.004) |
| Marital status (age 36) (ref = married) | | |  |  |  |  |
| Not married |  |  | 0.000 |  |  | .001 |
|  |  |  | (.015) |  |  | (.014) |
| Divorced |  |  | .058** |  |  | .073** |
|  |  |  | (.022) |  |  | (.027) |
| Widowed |  |  | .150 |  |  | -.072 |
|  |  |  | (.106) |  |  | (.278) |
| Any hospitalization (ages 34-36) | |  | .170*** |  |  | .128*** |
|  |  |  | (.019) |  |  | (.023) |
| Constant | .545*** | .546*** | .562*** | .387*** | .387*** | .455*** |
|  | (.007) | (.010) | (.029) | (.007) | (.011) | (.034) |
| *^a^*An estimate for parental income weight larger than .5 indicates origin is more salient than destination among mobile individuals. *Standard errors are in parentheses, *** p<.001, ** p<.010, * p<.050* | | | | | | |

Supplementary Table S3 Psychotropic drug dispensation and outpatient care (2005-2019) by social (n=11,199) with different versions of hospitalization – Diagonal reference model with linear link

|  | Women (N=5,903) | | Men (N=5,296) | |
| --- | --- | --- | --- | --- |
|  | Base model | Fully adjusted | Base model | Fully adjusted |
| Stable low income | .047*** | .047*** | .068*** | .067*** |
|  | (.014) | (.013) | (.013) | (.013) |
| Stable middle income | -.013 | -.014 | -.045*** | -.044*** |
|  | (.017) | (.016) | (.013) | (.013) |
| Stable high income | -.034* | -.034* | -.023 | -.023 |
|  | (.014) | (.014) | (.014) | (.014) |
| Weights |  |  |  |  |
| Parental income (ages 10, 17) | .742 | .716 | .530*** | .530*** |
|  | (.502) | (.463) | (.148) | (.15) |
| Adult income (ages 37-48) | .258 | .284 | .470** | .470** |
|  | (.502) | (.463) | (.148) | (.15) |
| Social mobility (ref = stable income) | |  |  |  |
| Downward | .033 | .032 | .044* | .045* |
|  | (.031) | (.029) | (.019) | (.019) |
| Upward | -.028 | -.026 | -.043* | -.043* |
|  | (.032) | (.03) | (.018) | (.018) |
| Single parent household (age 11) | .005 | .006 | .010 | .011 |
|  | (.022) | (.022) | (.024) | (.024) |
| Cognitive ability (age 13) | -.001 | -.001 | -.001 | -.001 |
|  | (.000) | (.000) | (.000) | (.000) |
| Social skills (age 13) | -.002 | -.002 | -.007 | -.007 |
|  | (.004) | (.004) | (.004) | (.004) |
| Marital status (age 36) (ref = married) | |  |  |  |
| Not married | .005 | .005 | .008 | .009 |
|  | (.015) | (.015) | (.014) | (.014) |
| Divorced | .060** | .059** | .060* | .063* |
|  | (.021) | (.021) | (.027) | (.027) |
| Widowed | .103 | .098 | -.108 | -.111 |
|  | (.105) | (.105) | (.277) | (.277) |
| Any hospitalization (ages 16-36) | .127*** |  | .114*** |  |
|  | (.013) |  | (.013) |  |
| Any hospitalization (ages 19-36) |  | .132*** |  | .115*** |
|  |  | (.013) |  | (.014) |
| Constant | .523*** | .522*** | .426*** | .409*** |
|  | (.030) | (.030) | (.035) | (.034) |
| *^a^*An estimate for parental income weight larger than .5 indicates origin is more salient than destination among mobile individuals. *Standard errors are in parentheses, *** p<.001, ** p<.010, * p<.050* | | | | |

Supplementary Table S4 Psychotropic drug dispensation (2005-2019, ages 52-66) by social mobility (n=11,199) – Diagonal reference model with logit link

|  | Women (N=5,903) | | | Men (N=5,296) | | |
| --- | --- | --- | --- | --- | --- | --- |
|  | Base model | Mobility adjusted | Fully adjusted | Base model | Mobility adjusted | Fully adjusted |
| Stable low income | .267*** | .255*** | .185** | .346*** | .383*** | .291*** |
|  | (.050) | (.055) | (.059) | (.058) | (.054) | (.058) |
| Stable middle income | -.071 | -.041 | -.030 | -.085 | -.194*** | -.196*** |
|  | (.048) | (.063) | (.070) | (.050) | (.058) | (.056) |
| Stable high income | -.196*** | -.213*** | -.155** | -.261*** | -.189*** | -.095 |
|  | (.048) | (.051) | (.054) | (.044) | (.055) | (.058) |
| Weights^a^ |  |  |  |  |  |  |
| Parental income (ages 10, 17) | .300** | .756 | .809 | .029 | .501*** | .565*** |
|  | (.107) | (.394) | (.621) | (.121) | (.143) | (.147) |
| Adult income (ages 37-48) | .700*** | .244 | .191 | .971*** | .499*** | .435** |
|  | (.107) | (.394) | (.621) | (.121) | (.143) | (.147) |
| Social mobility (ref = stable) | | |  |  |  |  |
| Downward |  | .159 | .142 |  | .207* | .212** |
|  |  | (.135) | (.150) |  | (.090) | (.080) |
| Upward |  | -.162 | -.135 |  | -.211* | -.195* |
|  |  | (.137) | (.152) |  | (.089) | (.081) |
| Single parent household (age 11) | |  | .029 |  |  | .069 |
|  |  |  | (.093) |  |  | (.103) |
| Cognitive ability (age 13) |  |  | -.003* |  |  | -.004* |
|  |  |  | (.002) |  |  | (.002) |
| Social skills (age 13) |  |  | -.008 |  |  | -.032* |
|  |  |  | (.016) |  |  | (.016) |
| Marital status (age 36) (ref = married) | | |  |  |  |  |
| Not married |  |  | .020 |  |  | .029 |
|  |  |  | (.060) |  |  | (.062) |
| Divorced |  |  | .266** |  |  | .271* |
|  |  |  | (.090) |  |  | (.114) |
| Widowed |  |  | .644 |  |  | -.3080 |
|  |  |  | (.462) |  |  | (1.23) |
| Any hospitalization (ages 34-36) | |  | .767*** |  |  | .600*** |
|  |  |  | (.082) |  |  | (.093) |
| Constant | .186*** | .188*** | .279* | -.454*** | -.452*** | -.186 |
|  | (.026) | (.042) | (.121) | (.028) | (.045) | (.145) |
| AIC | 8106.471 | 8107.935 | 8010.126 | 7012.665 | 7011.559 | 6966.912 |
| BIC | 8133.204 | 8148.034 | 8097.008 | 7038.963 | 7051.008 | 7052.384 |
| *^a^*An estimate for parental income weight larger than .5 indicates origin is more salient than destination among mobile individuals. *Standard errors are in parentheses, *** p<.001, ** p<.010, * p<.050* | | | | | | |

Supplementary Table S5 Psychotropic drug dispensation (2005-2019, ages 52-66) by social mobility (n=98,717) in the national cohort born in 1953 – Diagonal reference model with linear link

|  | Women (N=48,240) | | | Men (N=50,477) | | |
| --- | --- | --- | --- | --- | --- | --- |
|  | Base model | Mobility adjusted | Fully adjusted | Base model | Mobility adjusted | Fully adjusted |
| Stable low income | .051*** | .051*** | .045*** | .051*** | .052*** | .043*** |
|  | (.004) | (.004) | (.004) | (.004) | (.004) | (.004) |
| Stable middle income | -.010*** | -.011*** | -.007* | -.015*** | -.02*** | -.018*** |
|  | (.003) | (.003) | (.003) | (.003) | (.004) | (.004) |
| Stable high income | -.040*** | -.040*** | -.038*** | -.036*** | -.033*** | -.026*** |
|  | (.004) | (.004) | (.004) | (.003) | (.004) | (.004) |
| Weights^a^ |  |  |  |  |  |  |
| Parental income (ages 10, 17) | -.154* | -.054 | -.055 | -.306*** | -.049 | -.069 |
|  | (.070) | (.133) | (.152) | (.078) | (.124) | (.151) |
| Adult income (ages 37-48) | 1.154*** | 1.054*** | 1.055*** | 1.306*** | 1.049*** | 1.069*** |
|  | (.070) | (.133) | (.152) | (.078) | (.124) | (.151) |
| Social mobility (ref = stable) | | |  |  |  |  |
| Downward |  | -.001 | -.001 |  | .018* | .018* |
|  |  | (.009) | (.010) |  | (.009) | (.009) |
| Upward |  | -.014 | -.015 |  | -.016 | -.016* |
|  |  | (.009) | (.009) |  | (.008) | (.008) |
| Marital status (age 36) (ref = married) | | |  |  |  |  |
| Not married |  |  | .021*** |  |  | .016*** |
|  |  |  | (.005) |  |  | (.005) |
| Divorced |  |  | .122*** |  |  | .065*** |
|  |  |  | (.008) |  |  | (.009) |
| Widowed |  |  | .054 |  |  | -.060 |
|  |  |  | (.035) |  |  | (.061) |
| Any hospitalization (ages 34-36) | |  | .124*** |  |  | .120*** |
|  |  |  | (.007) |  |  | (.007) |
| Constant | .508*** | .513*** | .480*** | .485*** | .485*** | .482*** |
|  | (.002) | (.004) | (.004) | (.005) | (.005) | (.005) |
| *^a^*An estimate for parental income weight larger than .5 indicates origin is more salient than destination among mobile individuals. *Standard errors are in parentheses, *** p<.001, ** p<.010, * p<.050* | | | | | | |

| Supplementary Table S6 Psychotropic drug group^a^ specific analysis (2005-2019, ages 52-66) by social mobility (n=11,199) - Fully adjusted diagonal reference models with linear link | | | | | | | | |
| --- | --- | --- | --- | --- | --- | --- | --- | --- |
|  | Women (N = 5,903) | | | | Men (N = 5,296) | | | |
|  | N05A | N05B | N05C^c^ | N06A | N05A | N05B | N05C | N06A |
| Stable low income | .039*** | .023** | .013 | .053*** | .043*** | .041*** | .058*** | .057*** |
|  | (.006) | (.009) | (.011) | (.012) | (.006) | (.009) | (.012) | (.011) |
| Stable mid income | -.016** | .003 | 0 | .007 | -.023*** | -.016* | -.051*** | -.024* |
|  | (.006) | (.007) | (.002) | (.011) | (.006) | (.008) | (.012) | (.012) |
| Stable high income | -.023*** | -.026* | -.014 | -.061*** | -.02*** | -.024** | -.007 | -.033** |
|  | (.006) | (.01) | (.011) | (.012) | (.006) | (.009) | (.013) | (.012) |
| Weights^b^ |  |  |  |  |  |  |  |  |
| Parental income  (ages 10,17) | .348* | -.343 | 3.193 | .692* | .223 | .803** | .420*** | .582* |
|  | (.167) | (.743) | (2.531) | (.28) | (.138) | (.248) | (.125) | (.23) |
| Adult income  (ages 37-48) | .652*** | 1.343 | -2.193 | .308 | .777*** | .197 | .580*** | .418 |
|  | (.167) | (.743) | (2.531) | (.28) | (.138) | (.248) | (.125) | (.23) |
| Social mobility (ref = stable) | |  |  |  |  |  |  |  |
| Downward | .003 | -.031 | .070* | .027 | .000 | .035* | .015 | .043* |
|  | (.01) | (.025) | (.028) | (.025) | (.009) | (.015) | (.016) | (.02) |
| Upward | -.017 | .012 | -.061* | -.047 | -.013 | -.038* | -.034* | -.046* |
|  | (.010) | (.025) | (.028) | (.025) | (.009) | (.015) | (.016) | (.019) |
| Single parent household (age 11) | | |  |  |  |  |  |  |
|  | .004 | -.004 | .005 | .026 | -.008 | -.034* | .010 | -.002 |
|  | (.01) | (.018) | (.022) | (.021) | (.01) | (.016) | (.022) | (.02) |
| Cognitive ability (age 13) | |  |  |  |  |  |  |  |
|  | .000 | -.001** | -.001* | -.001 | .000 | -.001** | -.001 | .000 |
|  | (.000) | (.000) | (.000) | (.000) | (.000) | (.000) | (.000) | (.000) |
| Social skills (age 13) | |  |  |  |  |  |  |  |
|  | -.001 | .002 | -.001 | -.002 | -.003 | -.008** | -.01** | -.006* |
|  | (.002) | (.003) | (.004) | (.004) | (.002) | (.003) | (.003) | (.003) |
| Marital status (age 36) (ref = married) | | |  |  |  |  |  |  |
| Not married | .028*** | .015 |  | .006 | .025*** | .021* | -.004 | .010 |
|  | (.007) | (.012) |  | (.014) | (.006) | (.01) | (.013) | (.012) |
| Divorced | .025** | .045* |  | .087*** | .014 | .013 | .067** | .035 |
|  | (.010) | (.017) |  | (.020) | (.012) | (.018) | (.025) | (.023) |
| Widowed | .046 | .172* |  | .226* | -.025 | -.145 | .042 | .109 |
|  | (.048) | (.085) |  | (.099) | (.119) | (.188) | (.258) | (.236) |
| Any hospitalization (ages 34-36) | | |  |  |  |  |  |  |
|  | (.009) | (.015) |  | (.018) | (.009) | (.015) | (.021) | (.019) |
| Constant | .051*** | .240*** | .463*** | .353*** | .016 | .194*** | .357*** | .238*** |
|  | (.013) | (.024) | (.028) | (.028) | (.014) | (.023) | (.031) | (.029) |
| ^a^N05A neuroleptics, N05B anxiolytics, N05C sedatives, N06A antidepressants ^b^An estimate for parental income weight larger than .5 indicates origin is more salient than destination among mobile individuals.  ^c^Model did not converge with adjustment for marital status and hospitalization.  *A Standard errors are in parentheses, *** p<.001, ** p<.01, * p<.05* | | | | | | | | |

| Supplementary Table S7 Psychotropic drug dispensation (2005-2019) stratified by parental and adult income (n=11,199) | | | | | |
| --- | --- | --- | --- | --- | --- |
|  | Women  (N=5,903) | Men  (N=5,296) |  | Women  (N=5,903) | Men  (N=5,296) |
|  | Low parental income | |  | Low adult income | |
| Adult income - Low (ref) |  |  | Parental income - Low (ref) |  |  |
| Adult income - Middle | -.066** | -.089*** | Parental income - Middle | -.012 | -.041 |
|  | (.026) | (.027) |  | (.026) | (.028) |
| Adult income - High | -.055 | -.112*** | Parental income - High | -.049 | .010 |
|  | (.029) | (.032) |  | (.028) | (.030) |
|  |  |  |  |  |  |
|  | Middle parental income | |  | Middle adult income | |
| Adult income - Low | .069 | .090*** | Parental income - Low | .009 | .033 |
|  | (.028) | (.028) |  | (.027) | (.027) |
| Adult income - Middle (ref) |  |  | Parental income - Mid (ref) |  |  |
| Adult income - High | -.021 | -.025 | Parental income - High | .029 | .068* |
|  | (.028) | (.028) |  | (.028) | (.030) |
|  |  |  |  |  |  |
|  | High parental income | |  | High adult income | |
| Adult income - Low | .029 | .113*** | Parental income - Low | .040 | -.022 |
|  | (.027) | (.031) |  | (.029) | (.029) |
| Adult income - Middle | .038 | .051 | Parental income - Mid | .013 | -.040 |
|  | (.027) | (.029) |  | (.027) | (.026) |
| Adult income - High (ref) |  |  | Parental income - High (ref) |  |  |
| All models adjusted for single parent household, cognitive ability, social skills, marital status, and hospitalization*.*  *Standard errors are in parentheses, *** p<.001, ** p<.010, * p<.050* | | | | | |
